# Gender differences in examination behavior of 4th grade medical students: Results from an observational study on ECG e-learning

**DOI:** 10.1007/s00508-021-01959-z

**Published:** 2021-10-20

**Authors:** Noemi Pavo, Thomas Niedermaier, Stefanie Seitz, Harald Jäger, Jeanette Strametz-Juranek, Anita Rieder, Anahit Anvari-Pirsch

**Affiliations:** 1 Department of Internal Medicine II, Clinical Division of Cardiology, Medical University of Vienna, Währinger Gürtel 18–20, 1090 Vienna, Austria; 2 IT-Systems&Communications, Medical University of Vienna, Vienna, Austria; 3 Assessment&Skills, Medical University of Vienna, Vienna, Austria; 4 Study Department, Medical University of Vienna, Vienna, Austria; 5 Center of Rehabilitation—Bad Tatzmannsdorf, Bad Tatzmannsdorf, Austria; 6 Centre of Public Health, Medical University of Vienna, Vienna, Austria

**Keywords:** E‑learning, Electrocardiogram, Personality traits, Academic performance, Medical education

## Abstract

**Background:**

Computer-assisted teaching is becoming increasingly more important to acquire new knowledge and skills in medical curricula. The consequence of gender-characteristic personality traits on academic performance in e‑learning examinations are difficult to forecast. This study investigated gender-related differences in examination behavior among undergraduate medical students taking a web-based quiz.

**Methods:**

A total of 1315 4th grade medical students at the Medical University of Vienna completing the compulsory online moodle-based ECG quiz 2017/2018 were enrolled into this observational study. Individual data of examination behavior and quiz results as well as results of the final annual exam were extracted. Students were grouped into 10 strata according to academic performance. Variables between both sexes were compared using a nonparametrical test. Examination variables were correlated to performance.

**Results:**

Of the total study population 686 (52%) were female and 629 (48%) were male. The time until the first attempt and number of attempts performed was comparable between both sexes, however female students spent more time on the first attempt compared to their male colleagues (1592 sec [Q1–Q3: 999–2536] vs 1405 sec [Q1–Q3: 828–2395], *p* = 0.002), suggesting a higher self-discipline and risk-aversity. There was no difference regarding quiz scores or final ECG examination scores between female and male students (*p* = 0.869 and *p* = 0.396). Students who accessed the quiz earlier and less time spent for the first attempt tended to perform better at the final examination (r_s_ = 0.20, *p* < 0.001 and r_s_ = −0.15, *p* < 0.001).

**Conclusions:**

Gender-related differences in examination behavior already described for nononline based examinations are similarly observable in e‑learning. For this test, gender-immanent traits seem not to twist final examination results and impact academic performance.

## Introduction

The incorporation of gender-specific knowledge into medical curricula is imperfect due to the lack of evidence-based data on appropriate teaching strategies and material. Several studies have shown that male and female individuals, particularly medical students, exhibit different learning styles [1]. Moreover, there is evidence for gender-related differences in examination behavior, with female students showing more self-discipline whereas at the same time being less self-confident and more risk-averse [2]. It lies in the responsibility of the curricula and the teachers to address these differences to be able to develop and offer suitable teaching/learning methods. This question must especially be addressed by the academic community to provide high quality education equally promoting male and female students.

Computer-assisted teaching (CAT) and web-based learning bear an incredible opportunity by reaching large and heterogeneous audiences. The use of CAT becomes more and more important to acquire new knowledge and skills in medical curricula [3]. Online classes and number of universities offering pure online or hybrid blended online courses are growing considerably [4, 5]. While the strengths of the method are evident, it also brings many challenges. Besides the lack of social interaction and communication, which could result in lower motivation, individualities as different affinities towards computer use and acceptance could also influence teaching outcomes. While e‑learning has generally been regarded as a tool for “degendering”, gender-related differences seem to exist in many dimensions, e.g. preferred type of tasks or communication. In order to assure an equal benefit for both male and female students, e‑learning programs should continuously be evaluated to assess their impact on both genders. Especially in the setting of online examinations the consequence of gender-characteristic personality traits are difficult to forecast.

The aim of this study was to investigate gender-related differences in examination behavior among undergraduate medical students in an e‑learning-based examination.

## Material and methods

### Study population

All 4th grade medical students of the Medical University of Vienna taking the compulsory online Moodle-based electrocardiograph (ECG) quiz in 2017 and 2018 were enrolled into this observational study. Students who have never logged into the web system were excluded from the analysis. The study protocol was approved by the data safety committee of the Medical University of Vienna.

### Characteristics of the ECG online quiz

The online ECG quiz was implemented in the year 2016 as part of the ECG blended learning module of the Medical University of Vienna. The quiz is compulsory and takes place between the ECG lecture and ECG practice element intending to optimally prepare the students for the practice element. The test is open for 3 weeks whereas students are notified well in advance and a reminder is sent out 3 days before the deadline. In isolated cases an extension of the deadline may be allowed. The test consists of 15 randomly selected multiple-choice questions out of 146 with both single choice and multiple answer options. The quiz may be repeated up to 3 times whereas a positive result is obtained in the case of answering 12 questions correctly. There is no time limit for completing the quiz.

### Data

Demographic data as age, gender and time of inscription were assessed. Individual data of the quiz results were extracted from the Moodle platform. In detail, date of attempts and time until the first attempt, the number of total attempts, duration of each attempt as well as final quiz results were documented. Additionally, the results of the corresponding final examination (objective structured clinical examination [OSCE]) within the same study year were collected, i.e. total score and score at the ECG station of the OSCE parcours.

### Statistical analysis

Continuous variables were presented as median and the interquartile range as quartile 1 and quartile 3 (IQR as Q1–Q3) and categorical data as counts and percentages. Descriptive statistics were used to describe the baseline characteristics of the study population. Baseline characteristics between both genders were compared. To investigate gender-related differences in examination behavior, the time until first attempt, duration of first attempt as well as quiz results and final examination results concerning ECG were compared between female and male students. For all comparisons nonparametrical tests were used for continuous data, i.e. the Mann-Whitney U‑test, and Fisher’s exact test for categories. In order to investigate the relationship of examination behavior and educational performance, students were divided according to final examination scores into groups and Spearman’s correlation coefficient was calculated for the quiz-related variables with groups. For all tests two-sided *p*-values lower 0.05 were considered to indicate statistical significance. No adjustment for multiple testing has been performed as *p*-values are of explorative nature.

## Results

### Demographic data

A total of 1315 4th grade medical students were included into the present study, 686 (52%) female and 629 (48%) male students. Baseline characteristics of the study population are displayed in Table 1. Male students were older (23 years, Q1–Q3: 22–25 years vs. 23 years, Q1–Q3: 22–24 years, *p* < 0.001, as mean ± standard deviation STD 24 ± 3 vs. 23 ± 3) and inscribed later after the school leaving examination by approximately 1 year (63 months, Q1–Q3: 52–76 months vs. 52 months, Q1–Q3: 51–64 months, *p* < 0–001), corresponding to the obligatory service in the Federal Armed Forces or social service for males in Austria. There were no significant differences regarding the months spent studying.

**Table 1:** Baseline characteristics of 4th grade medical students taking the compulsory e‑learning ECG examination. Variables are listed as medians and interquartile ranges as Q1–Q3. Differences between female and male students were compared using the Mann-Whitney U‑test

Variable	Total cohort (*n* = 1315)	Female students (*n* = 686)	Male students (*n* = 629)	*p*-value
Age, years (Q1–Q3)	23 (22–24)	23 (22–24)	23 (22–25)	**<0.001**
Time since school-leaving examination, months (Q1–Q3)	52 (51–75)	52 (51–64)	63 (52–76)	**<0.001**
Study time, months (Q1–Q3)	37 (37–38)	37 (37–38)	37 (37–38)	0.652
Time until first attempt, days (Q1–Q3)	15 (8–23)	14 (8–12)	16 (7–24)	0.096
Time elapsed at first attempt, s (Q1–Q3)	1506 (905–2476)	1592 (999–2536)	1405 (828–2395)	**0.002**
ECG quiz results, score (Q1–Q3)	14 (13–14)	14 (13–14)	14 (13–15)	0.869
Final examination ECG results, score (Q1–Q3)	24 (22–25)	24 (22–25)	24 (22–25)	0.396

Bold type indicates statistical significance

### Characteristics of the online ECG quiz

The time until first attempt after setting up definitive connection for students was 15 days (Q1–Q3: 8–23 days) and students took 1506 s (Q1–Q3: 905–2476s) corresponding to nearly 25 min for the completion of the first attempt. Most students with 1076 (82%) required 1 attempt, 189 (14%) and 50 (4%) students required 2 or 3 attempts for the completion of the quiz, respectively. The time spent per attempt decreased with every new attempt (1506 s, Q1–Q3: 905–2476s vs. 1272 s, Q1–Q3: 705–2352s vs. 1184 s, Q1–Q3: 674–2699s, *p* = 0.009 for comparison between all groups).

### Gender-related differences in e-learning examination behavior

**Fig. 1:**
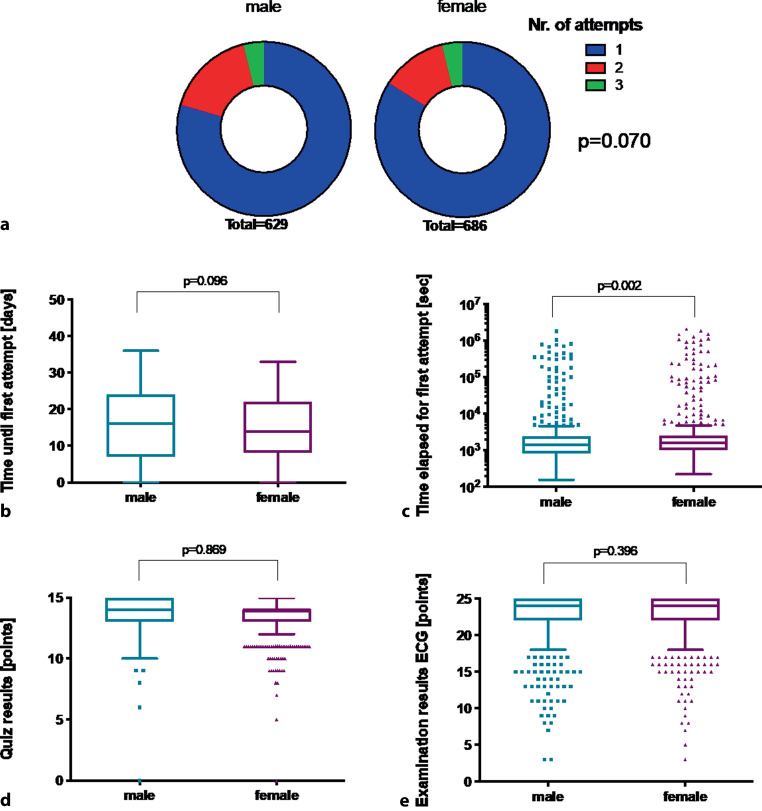
Gender-related differences in examination behavior in ECG e‑learning. **a** Number of attempts, **b** time until first attempt, **c** time elapsed for first attempt as well as the **d** quiz results and **e** final examination results are shown for female and male students. Medians were compared by the Mann-Whitney U‑test

The comparison of examination variables between genders are graphically displayed in Fig. 1. The time until the first attempt was comparable between both sexes (16 days [Q1–Q3: 7–24] for males vs 14 days [Q1–Q3: 8–12] for females, *p* = 0.096); however, female students spent more time on the first attempt compared to their male colleagues (1592 sec [Q1–Q3: 999–2536] vs 1405 sec [Q1–Q3: 828–2395], *p* = 0.002). There was a trend towards a higher total number of attempts for male students (576 [84%], 84 [12%] and 26 [4%] for females vs 500 [79%], 105 [17%] and 24 [4%] for males completing 1, 2 or 3 attempts, *p* = 0.070). There was no significant difference regarding ECG quiz results and final ECG examination scores between female and male students (*p* = 0.869 and *p* = 0.396).

### Examination behavior according to educational performance

**Fig. 2:**
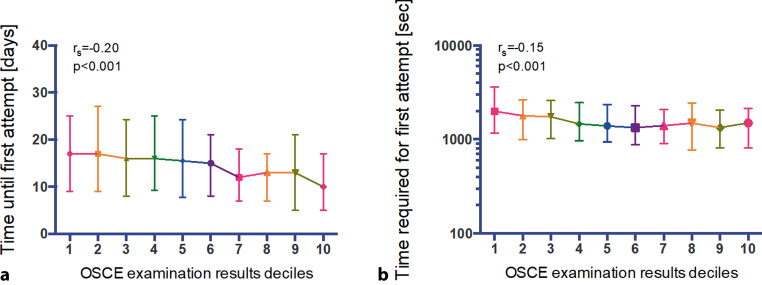
E‑learning examination behavior according to educational performance. **a** Time until first attempt and **b** time required for the first attempt are shown for different groups with increasing educational performance. Median and Q1–Q3 are plotted for deciles of educational performance for the time until first attempt and the time required for the first attempt, the Spearman Rho’s correlation coefficient is indicated for the correlation of the respective variables and the groups. *OSCE* Objective Structured Clinical Examination

Fig. 2 shows the results for the analysis of examination behavior according to educational performance. Both the time until first attempt as well as the time used for the first attempt were significantly lower for students with increasing scores at the final examination (r_s_ = −0.20, *p* < 0.001 and r_s_ = −0.15, *p* < 0.001) (Fig. 2). There was also a trend to lesser number of attempts needed with higher educational performance (*p* = 0.037).

## Discussion

This is the first report on gender-related differences in examination behavior concerning an elaborate e‑learning tool. Baseline characteristics of female and male students were mainly comparable. The time until the first attempt and number of attempts performed was comparable between both sexes; however, female students spent more time on the first attempt compared to their male colleagues. There was no difference regarding ECG quiz scores or final ECG examination scores between female and male students. Students who accessed the quiz earlier and spent less time for the first attempt generally performed better at the final examination.

There is a great demand for more flexible and convenient methods for obtaining a higher education. Technological advances have made online learning tools available and both economical and practical. As a consequence, online classes and number of universities offering pure online or hybrid blended online courses are growing considerably [4, 5]. Online learning may be defined as instruction delivered electronically via the internet, intranet, or multimedia platforms [6]. Online learning may be delivered either synchronously, where the instructor and students must be online simultaneously, or asynchronously, where there are no time restrictions and students and teachers do not have to be online at the same time [7]. Online technology can enable greater student reflection and foster more thoughtful and responsible comments than what might occur in face-to-face classrooms. Moreover, it offers more flexibility and convenience, makes it easier to work in collaborative groups and schedule meetings [8]. It allows control over content, learning sequence, pace of learning, time, and media resulting in a personalized and self-organized approach allowing the learning objectives to be more effectively met [9]. While the strengths of the method are evident, it also brings many challenges. A study found that it is more difficult to provide affective support to students in online learning, where affective support was defined as “communications from instructors to students that the students are important and valued individuals” [10]. In another study, the lack of social interaction was identified as the single most important barrier to students learning online. Administrative and instructor issues, time and support for studies, and learner motivation were additional significant barriers [11]. In order to deliver high quality and equitable online learning approaches, instructors and designers need a better understanding of how students perceive online learning tools.

Several studies have showed that gender-related differences regarding individual practice, acceptance, and frequency on the use of online learning tools seem to be lacking, consequently online learning has also been embraced as a tool of “degendering” [12]. On the other hand, gender differences in the attitude towards technology use have long been a concern in education. A recent meta-analysis indicated that males still hold more favorable attitudes towards technology use, albeit probably with small effect sizes [13]. Although technology use has increased exponentially within the last 20 years there is only a minimal reduction in the gender attitudinal gap in general [13]. Other data point out differences in computer skills and problem-solving abilities. Generally, males seem to have more positive attitudes towards computer use, have greater experience with computers and use computers much more frequently than females [14]. Additionally female and male students seem to prefer different type of tasks, teaching tools, lay-out and types of communication.

In experimental literature, women have been found to be more risk-averse on average, less willing to compete, and less overconfident than men [15]. These gender differences in preferences and personality traits are thought to explain a significant proportion of gender gaps in the labor market [2]. Noncognitive traits, such as self-discipline, can explain the fact that women typically receive higher grades than predicted by their performance on ability or achievement tests, and men receive lower than expected grades [16]. Despite these hints from experimental settings, there is a striking lack of research on gender-related differences in competitive environments of real-life settings. In one of the few studies female students at the Stockholm Economic School were more likely to take voluntary initial quizzes and attain seminar boni on the final exam by near perfect seminar attendance to increase their grades in a course; however, they were less likely to retake quiz questions in the final examination, which could alter their established score [2]. This indeed suggests that female students show more self-discipline but are more risk-averse and less overconfident than their male counterparts. These anticipated differences in examination behavior may have important implications in terms of final grades at academic institutions. This highlights the need for a gender-related assessment of academic examination strategies, especially every new e‑learning measure and program in order to assure equability.

After the announcement of the quiz, the time until start of the first attempt was comparable for both female and male students. This variable could equally reflect self-discipline, motivation and curiosity; however, female students spent more time on the first attempt. This suggests a similar behavior as found in the Swedish study with females showing more self-discipline and being more risk-averse, not willing to waste one attempt out of three by submitting answers quickly without checking them. There was a trend towards an increased number of attempts for male students compared to females, which similarly could reflect the gender-specific personality traits described above. Most importantly, gender-related traits do not seem to influence final grades, quiz scores and scores of the final ECG examination were comparable between both sexes. Overall performance, reflected by final examination grades, was significantly inversely associated to the time until the first attempt and the time spent for the first attempt of the ECG quiz, proposing that high-performing students are more characterized by a prompt access to the quiz and are fast quiz takers spending less time on questions.

The present study investigated gender differences among medical students in an academic setting performing an e‑learning based examination. The set-up involved risk and judgment about own ability but was otherwise noncompetitive in the sense that students could perform the quiz in a comfortable environment without pressure to perform and personal distress. At the time of analysis the students had already completed the test and therefore were not influenced by the study. Due to the retrospective design, it was not possible to assess individual personality traits and to relate or adjust the particular findings to such characteristics. Overall examination results were very good and it can be assumed that the impact of personality traits varies with the level of difficulty and performance pressure within examination situations.

The data contribute to the literature on gender differences in behavior in several ways. First, the study analyzed a real-life academic setting providing relevant information for the persons responsible for planning teaching strategies in higher education. Moreover, the first time gender differences regarding an e‑learning-based examination were studied highlighting that i) gender differences are indeed observable in this form of teaching instrument yet ii) the depicted differences in behavior might not influence the final ratings of academic performance. In general, the observed differences in examination behavior regarding the investigated e‑learning tool seem to be slight. As final examination results are not influenced by the preceding e‑learning tool the equal opportunity concept can be regarded as fulfilled concerning gender. Nevertheless, the results may not generalized to other e‑learning measures with different designs and notably another level of difficulty and pressure to perform, which could enhance gender-related differences above attitudes toward technology. It is important for medical curricula to screen various components of the educational program to filter out possible gender-specific issues and to evaluate and survey new online learning programs in order to have a better understanding of students perception and performance.

## Conclusion

The results show that gender-related differences already described for nononline based examinations are similarly observable in e‑learning-based testing. At least in this particular case, gender-immanent traits do not seem to twist final examination results and impact academic performance.

## Limitations

This was a single-center experience of the largest Medical University in Austria thereby representative for medical students but not for students in other fields. Further studies with different e‑learning tools, modalities as well as the inclusion of multiple sites need to be conducted to further characterize gender-related behavior in computer assisted learning in order to assess more generalizable results. In our setting, complete separation between different possible mechanisms, such as risk aversion, overconfidence, self-discipline or procrastination may evidently not be depicted.
